# A survey of AI ethics in business literature: Maps and trends between 2000 and 2021

**DOI:** 10.3389/fpsyg.2022.1042661

**Published:** 2022-12-19

**Authors:** Marco Tulio Daza, Usochi Joanann Ilozumba

**Affiliations:** ^1^Institute of Data Science and Artificial Intelligence (DATAI), School of Economics and Business, University of Navarra, Pamplona, Spain; ^2^Information Systems Department, University Center for Economic and Administrative Sciences (CUCEA), University of Guadalajara, Guadalajara, Mexico; ^3^HHL Leipzig Graduate School of Management, Leipzig, Germany; ^4^Wittenberg Center for Global Ethics, Wittenberg, Germany

**Keywords:** ethical theories, algorithmic bias, privacy, transparency, work, automation, social media

## Abstract

Artificial intelligence is spreading rapidly in business products and processes, with innovations that bring great benefits to society; however, significant risks also arise. AI-enabled systems make decisions autonomously and influence users and the environment, presenting multiple ethical issues. This work focuses on the ethics of AI use in business. We conduct a survey of business journal articles published between 2000 and mid-2021 to identify the most influential journals, articles, and authors, the most influential ethical schools, and the main ethical issues of AI in business. It describes the state-of-the-art in the field and identifies trends in ethical issues arising from AI. Thus, we present maps and trends of the ethics in AI in business literature.

## Introduction

The availability of massive datasets and new machine learning techniques has triggered rapid advances in AI in the past decade (Acemoglu et al., 2022). This technology-driven transformation is reshaping business, economy, and society (Loureiro et al., 2021). Innovations bringing great benefits and new challenges herald the arrival of a new industrial revolution (Marsh, 2012). Therefore, significant risks arise, and with them, the need for ethical assessment.

The fourth industrial revolution is causing a dramatic transformation of the world economy (Schwab, 2017). Companies as diverse as Google, Spotify, Under Armor, and so forth enhance their performance through the adoption of AI (Vlačić et al., 2021). Corporations that provide these platforms, such as Microsoft, Amazon, Alphabet (Google), and Apple, form part of a group whose market capitalization has exceeded one trillion dollars.^1^ Worldwide spending on cognitive and AI systems has grown from $24.0 billion in 2018 (Loureiro et al., 2021) to $93.5 billion in 2021 (Zhang et al., 2022).

The impact of AI is not limited to business and the economy; it prompts a profound transformation of work (Rodriguez-Lluesma et al., 2020). Like previous industrial revolutions, the fourth raises concerns that automation will wipe out jobs (Autor, 2015). AI-driven robots are replacing blue-collar workers in factories (Belanche et al., 2020), while Robotic Process Automation (RPA) systems are taking white-collar jobs. AI-based platforms are writing essays (Knibbs, 2022), computer code (Thompson, 2022), and creating art (Johnson, 2022a).

According to a University of Oxford study, 47% of jobs will be lost due to automation in the next 25 years (Frey and Osborne, 2013). However, Beerbaum and Otto (2021) suggests that these jobs will soon be replaced by new ones. Nevertheless, it is unclear how quickly they can be recovered or if newly created jobs will be of quality. Companies in the *On-Demand Economy* fuel the proliferation of precarious jobs; for Cherry (2016), this has devalued work, driving wages below the legal minimum and providing an excuse to avoid paying social security benefits.

AI transformation of work has a broad social impact. AI-enabled systems determine whether someone is hired, promoted, or approved for a loan, as well as which ads and news articles consumers see (Martin, 2019). These algorithmic decisions can have unfair negative consequences or even violate human rights (Kriebitz and Lütge, 2020).

There are other harms originating from AI's development and deployment. Training data for machine learning is obtained and used in ways that often violate people's privacy (Thiebes et al., 2020). AI-enabled systems can be used for surveillance (Stahl et al., 2021). Social media platforms wield enormous influence on users. They can undermine public health (Bhargava and Velasquez, 2020), polarize social groups, affect democratic participation, foster the spread of fake news and conspiracy theories (Zuboff, 2018), and even aid in terrorist attacks (Rauf, 2021).

We must consider that the ability of humans to cause harm to others has increased with new technologies; now, machines themselves could cause damage (Letheren et al., 2020). Consequently, ethical assessment is required to understand AI-associated issues, support better decisions, and establish standards to develop and implement AI systems. Thus, AI could also serve to promote flourishing.

However, it is not enough to have an evaluation that sheds light on our actions (or that of the machines). It is also necessary to justify and convince the organization's leadership why we opt for specific behavior. This acquires relevance in business, even more so where ethical choices are not usually the most lucrative. Furthermore, problems may arise when there is no theoretical support in the face of complex ethical problems, such as the lack of supporting arguments, weak justifications, or erroneous decisions. Therefore, we believe that discussing ethical theories is essential.

The first motivation of our work is to understand the state of AI ethics in business publications from a perspective that recognizes its intrinsic moral value. We note a lack of research with a holistic perspective in the literature, which is essential to study this topic. We highlight three key aspects. First, we conducted a bibliometric analysis of the literature on the subject, identifying the most influential journals, articles, and authors, which allowed us to situate ourselves in the field. Second, we categorize the main ethical issues of AI in business and identify the schools of ethical thought that are being used to address them. This perspective is necessary to recognize the value of ethics as an inquiry tool to evaluate competing tech policy strategies and practices, which have been downplayed by the industry as a communication strategy or a facade to cover up unethical behaviors (Bietti, 2020).

Our second motivation is to provide a survey of AI ethics literature with a comprehensive approach focused on the field of business, including more than specific areas, functions, or principles. We intend to find gaps in the literature, identify under-researched areas, and map the state-of-the-art in the field.

Although current literature presents valuable insights into specific domains, no research article focuses on the issues of AI in the business field comprehensively using an ethical perspective. None of the eleven Systematic Literature Reviews (SLRs) published between 2000 and 2021 had AI ethics as the primary focus across all business areas and functions (see Table 1). Most SLRs are centered on AI topics in business or business ethics separately. Only two reviews have an ethical approach to AI in business. Although they address specific domains, Bhatta (2021) studies the digitalization of leadership, and Hermann (2021) explores AI in marketing. Ryan and Stahl (2021) carried out the only SLR focused on AI ethics. However, their work does not focus on the business domain and has a limited approach to ethics since its scope is limited only to principles and guidelines.

**Table 1:** Systematic literature reviews that address AI, business, and ethics between 2000 and 2021.

**Author(s)**	**Title**	**Year**	**Source**	**Scope**	**Main topic**	**Focus**
Bhatta et al.	Emerging ethical challenges of leadership in the digital era: A multi-vocal literature review	2021	Electronic Journal of Business Ethics and Organization Studies	1985–2020	Ethical challenges for leadership	Ethics of AI in business
Caner and Bhatti	A conceptual framework on defining business strategy for artificial intelligence	2020	Contemporary Management Research	2015–2019	AI business strategy	AI in business
Hermann et al.	Leveraging artificial intelligence in marketing for social good - an ethical perspective	2021	Journal of Business Ethics	No time constraints	Ethics of AI in marketing	Ethics of AI in business
Liu et al.	A big-data approach to understanding the thematic landscape of the field of business ethics, 1982–2016	2019	Journal of Business Ethics	1982–2016	Business ethics	Business ethics
Losbichler and Lehner	Limits of artificial intelligence in controlling and the ways forwards: A call for future accounting research	2021	Journal of Applied Accounting Research	No time constraints	AI in management accounting and monitoring	AI in business
Loureiro et al.	Artificial intelligence in business: State of the art and future research agenda	2021	Journal of Business Research	1970–2019	AI in business	AI in business
North-Samardzic	Biometric technology and ethics: Beyond security applications	2020	Journal of Business Ethics	No time constraints	Biometric technology and privacy in business	Business ethics
Ryan and Stahl	Artificial intelligence ethics guidelines for developers and users: Clarifying their content and normative implications	2021	Journal of Information, Communication and Ethics in Society	No time constraints	Ethic guidelines for AI	Ethics of AI
Schinagl and Shahim	What do we know about information security governance? “from the basement to the boardroom”: Toward digital security governance	2020	Information and Computer Security	No time constraints	Information security governance	Business
Syvänen and Valentini	Conversational agents in online organization-stakeholder interactions: A state-of-the-art analysis and implications for further research	2020	Journal of Communication Management	No time constraints	Chatbots in business	AI in business
Vlačić et al.	The evolving role of artificial intelligence in marketing: A review and research agenda	2021	Journal of Business Research	1987–2020	AI in marketing	AI in business

As a third motivation, we attempt to establish the connection between ethical schools of thought and the main AI issues in business. Thus, we classified papers into three leading ethical schools to measure their influence. Few authors study this phenomenon from the perspective of ethical theories, whether deontological, consequentialist, virtue ethics or a combination. Hermann (2021) carried out the only SLR that adopted an ethical theory standpoint, complementing deontological considerations with a utilitarian perspective. The other SLRs do not endorse an ethical theory. Most authors do not anchor their proposals in a foundational ethical theory. Some merely acknowledge that ethical problems exist and that future research is needed. Furthermore, we did not find an SLR or a research article that addresses the influence of ethical theories on the topic of AI in business.

Unlike most articles, which analyze AI ethics in an isolated context, this paper offers a survey of business journal articles focused comprehensively on AI ethics (not just guidance documents, Ryan and Stahl, 2021) across business domains and topics (not just leadership, Bhatta, 2021; marketing, Hermann, 2021; strategy, Caner and Bhatti, 2020) that connects the issues to specific ethical schools or theories. In this way, AI ethics connects not only to business ethics but also to socioeconomic and political ethics in general through major ethical traditions.

We organized this article into four sections. This introduction presents an outline of the impact of AI and our motivations. Section two continues with the methodology, the setting up of our database, and our research questions with the metrics and techniques used. Section three discusses our findings regarding the most influential articles, journals, and authors, presents a classification of the articles according to the ethical school used (if any), and proposes a categorization for the most recurring issues. We then proceed to analyze the evolution of these issues. Finally, in the fourth section, we present the maps and trends identified as conclusions and suggest areas for future research.

## Methodology

We built our dataset by performing a structured search for scientific articles that study the ethics of AI in business and management between 2000 and mid-2021. We used five major academic databases: Web of Science, Scopus, Emerald, Business Source Ultimate, and Google Scholar, from which we retrieved 349 articles using the search strategy shown in Table 2.

**Table 2:** Search strategy.

**The search strategy of documents from databases**	
***The inclusion and exclusion criteria for “journal articles only,” “English language only,” and years 2000–2021 were done before each of the searches took place***	
Web of Science (WOS)	(“Artificial Intelligence”) **AND** (ai) **AND** (“virtue ethics”) **OR** (ethics*) **AND** (business*) **OR** (“business management”) **40 journal articles**
Scopus	(“Artificial Intelligence”) **AND** (ai) **AND** (“virtue ethics”) **OR** (ethics*) **AND** (business*) **OR** (“business management”) **56 journal articles**.
Google Scholar	Search for articles with ALL words: “Business” With the exact phrase: “artificial intelligence” With at least one of the words: “virtue”/ “ethics”/ “virtue ethics” Where words occur: anywhere in the article Source: business ethics*161 articles
Business Source Ultimate (EBSCO)	“Artificial Intelligence” + “Ethics in Business” + “Ethics in Business Management” **38 articles**
Emerald Insight	“Artificial intelligence” **AND** Ethics*AND business Peer-reviewed Journals, Open-Access **54 articles**

After a screening process, we discarded duplicates, book chapters, and other irrelevant documents. The remaining articles were filtered to leave 95 articles in our primary dataset.

We gathered all the groups from different databases (SCOPUS, WOS, Google Scholar, EBSCO, and Emerald Insight), each with a different file format, into a single file and standardized its set-up. We used the CSV (comma-separated values) structured table format required by the VOSviewer software to build and visualize bibliometric networks. To complete our database, we then conducted an online search on authors' profiles, institutions, and countries. We also verified the citations of each article and those of each author with a cutoff date of May 11^th^, 2022.

We cleaned up the “keywords” column of our database file. This process was necessary to gain clarity and prevent the same concept from appearing under different names. We replaced all keyword occurrences of “AI,” “artificial intelligence (ai),” and “artificial intelligence” with “Artificial Intelligence”; additionally, we abbreviated all keywords that included “artificial intelligence” + “another word” (e.g., “artificial intelligence ethics,” “artificial intelligence safety,” “artificial intelligence guidelines”) to use “ai” + “another word.”

Finally, we proceeded with the formulation of research questions that would guide our work.

### Research questions

This study comprises five main research questions (hereafter referred to as RQ):

- RQ1: What are the most influential journals?
- RQ2: What are the most influential articles?
- RQ3: Which are the most influential authors?
- RQ4: What are the major schools of thought on the ethics of AI in business?
- RQ5: What are the main ethical issues of AI in business?

We carefully reviewed the 95 papers and applied bibliometric analysis techniques. Scholars use bibliometric analysis to uncover emerging trends in author, article, and journal performance, collaboration patterns, and research constituents and to explore the intellectual structure of a specific domain in literature (Donthu et al., 2021). This method encapsulates the application of several quantitative techniques to bibliometric data, such as using performance analysis indicators and science mapping techniques.

### Most influential articles (RQ1), journals (RQ2), and authors (RQ3): A performance analysis

For RQ1, RQ2, and RQ3, we used *performance analysis* techniques that examine the contributions of different research constituents using publication-related and citation-related metrics (Donthu et al., 2021). Using citations as a metric to identify the most influential publications allowed us to understand the intellectual dynamics of this research field (Donthu et al., 2021) and measure their impact and influence.

For RQ1, besides the journal's total citations, we contrasted the number of publications in the timeframe of this review to assess productivity.

For RQ2, we built graphics using the number of articles and total citations over time to analyze their evolution. We could not perform a *co-citation analysis* with the information gathered from multiple databases. The reason was that some did not provide complete metadata; information regarding references was also missing from some papers. Furthermore, the total number of authors, the institutions, and countries of origin of 5 articles were not identifiable with the articles, nor were they found in the searches carried out in academic databases.

For RQ3, we used the total number of citations*, h-index*, institution, and country to deepen the analysis of author influence. It is important to mention that citation does not necessarily mean agreement with an author; however, it could indicate the author's relevance to the discussion.

Of many performance indicators, we chose the *h-index* because it assumes that the number of citations received by a researcher is a better indicator of the relevance of their work than the number of papers they publish or journals where they published. It considers the number of papers published and the citations to those papers in a balanced way. Thus, it is helpful in making comparisons between scientists (Hirsch and Buela-Casal, 2014).

We finally examined the countries with most publications. Since articles are often published by multiple authors from different institutions, we considered each author's institution.

### Major ethical schools of thought: Screening literature (RQ4)

For RQ4, we turned to the Stanford Encyclopedia of Philosophy (SEP) for major ethical schools of thought, and we found that consequentialist, deontological, and virtue ethics are preferred by most authors in different domains (Mathieson, 2007; Moriarty, 2008; Hursthouse and Pettigrove, 2018; Norman, 2022). Therefore, we used them as a starting point in the field of AI in business.

To associate articles and authors with one or more ethical theories, we used SEP entries on Deontological Ethics (Larry and Moore, 2021), Consequentialism (Sinnott-Armstrong, 2021), and Virtue Ethics (Hursthouse and Pettigrove, 2018), yielding the following questions as criteria: (a) Are the solutions given to the ethical issues raised in the article derived from duty or a rule-based approach?, for deontologist approaches; (b) Are there references to outcomes, utility, or the primacy of consequential methods for establishing ethical principles? Does the argument involve calculating utility or benefits? for consequentialists; and (c) does the author suggest the approach of AI ethics from the standpoint of eudaimonia/flourishing? While tackling different ethical issues, are there references to virtues or virtuous agents? for virtue ethics.

We then proceeded to review the arguments in the publications and classify each into these categories. Some articles could have more than one ethical school perspective or not have any. After classifying the papers, we used the information collected and unified the databases to associate ethical theories with authors and publication dates.

### Main ethical issues (RQ5): Science mapping and inductive approach

We used science mapping techniques to identify the main issues in our topic and answer RQ5. These techniques examine how research constituents are connected and identify intellectual interactions and structural connections (Donthu et al., 2021).

The co-word analysis belongs to the science mapping toolbox. It is a technique that examines the actual content of the publication. This method assumes that words that frequently appear together have a thematic relationship with one another (Donthu et al., 2021). So, we applied this technique to identify the main thematic clusters in our dataset using the co-occurrence of keywords feature of the VOSviewer software.

The software identified that from 404 keywords set, there were 303 connected and forming a network, along with five thematic clusters. Some keywords can have a very general connotation (e.g., artificial intelligence, ethics), so it could be challenging to assign them to a thematic cluster (Donthu et al., 2021). Hence, we only used the most important concepts in this map as a supplementing resource.

Subsequently, through the review of our bibliographic set, we found ethical issues that repeatedly appear. Whether in developing or deploying AI-enabled systems, those issues arise across different business functions and industries. We took into account the article by Hermann (2021), in which he identifies transparency, justice and fairness, non-maleficence, responsibility, and privacy, as the most mentioned principles in the scientific literature on AI ethics.

We also looked at other sources for the most relevant ethical issues and concerns about AI in a general context (not just business). The “gray” literature, as opposed to “white” literature, is non-peer-reviewed scientific information that is not available using commercial information sources (Yasin et al., 2020). One fundamental feature of gray literature material is that it is readily published and often posted as soon as written (Vaska et al., 2010). Hence, we refer to the gray literature to contrast our findings (see Table 3).

**Table 3:** Main debates, principles, and concerns over AI ethics in gray literature.

**Level**	**Document**	**Issuer/Institution**	**Country/ Scope**	**Main debates, principles, and concerns**	**Year**
III	Asilomar AI Principles	Future of Life Institute	International	Safety, failure transparency, judicial transparency, responsibility, value alignment, human values, personal privacy, liberty and privacy, shared benefit, shared prosperity, human control, non-subversion, AI arms race, capability caution, importance, risks, recursive self-improvement, common good, research goal, research funding, science-policy link, research culture, race avoidance.	2017
I	General Data Protection Regulation (GDPR)	European Commission	European Union	Lawfulness, fairness, and transparency, purpose limitation, data minimization, accuracy, storage limitation, integrity and confidentiality, accountability	2018
III	Business Ethics and Artificial Intelligence	Institute of Business Ethics	UK	Accuracy, respect for privacy, transparency, interpretability, fairness, integrity, control, impact, accountability	2018
I	Ethics guidelines for trustworthy AI	High-level expert group on artificial intelligence. European Commission	European Union	Human agency and oversight, technical robustness and safety, privacy and data governance, transparency, diversity, non-discrimination and fairness, societal and environmental well-being, accountability	2019
II	AI Governance Principles	China's Ministry of Science and Technology	China	Harmony and friendliness, fairness and justice, inclusiveness and sharing, respect for privacy, security and controllability, shared responsibility, open cooperation, agile governance	2019
III	Ethics of Artificial Intelligence and Robotics	Stanford Encyclopedia of Philosophy	USA	Privacy and surveillance, manipulation of behavior, opacity of AI systems, bias in decision systems, human-robot interaction, automation and employment, autonomous systems, machine ethics, artificial moral agents, singularity	2020
III	The Oxford Handbook of Ethics of AI	Oxford Academic	UK	Fairness, accountability, transparency, responsibility, discrimination, future of work, AI as moral right-holder, AI as sentient, autonomy, algorithmic governance,	2020
IV	AI ethics	IBM	USA	Principles: Respect for persons, beneficence, justice. Concerns: Technological singularity, AI impact on jobs, privacy, bias and discrimination, accountability	2021
IV	Artificial Intelligence at Google: Our Principles	Google	USA	Be socially beneficial, avoid creating or reinforcing unfair bias, be built and tested for safety, be accountable to people, incorporate privacy design principles, uphold high standards of scientific excellence, be made available for uses that accord with these principles.	2021
II	Responsible Artificial Intelligence Strategy and Implementation Pathway	The Department of Defense (DoD) of the USA	USA	Responsible, equitable, traceable, reliable, governable	2022
III	The state of AI ethics. Volume 6	Montreal Institute of AI Ethics	Canada	Privacy, bias, social media and problematic information, AI design and governance, laws and regulations	2022
IV	Microsoft responsible AI principles	Microsoft	USA	Fairness, reliability and safety, privacy and security, inclusiveness, transparency, accountability	2022
II	US National AI Initiative Act of 2020	US Government	USA	Explainable AI, AI bias, and AI security.	Ongoing
II	Trustworthy and Responsible AI	National Institute of Standards and Technology (NIST)	USA	Accuracy, explainability and interpretability, privacy, reliability, robustness, safety, security (resilience), mitigation of harmful bias.	Ongoing

Level I, International organization; Level II, Government; Level III, Academic Institution; Level IV, Private Company.

We reviewed 14 documents and organized them into four levels according to their publishing instance. On the first level, we review international organizations; the second level governments; the third level academic institutions; and the fourth level private companies. This review identifies the same issues, central debates, and concerns as in scientific literature.

Finally, we use an inductive approach to identify the main debatable issues, concerns, and values. For example, transparency and confidentiality, along with concerns about privacy violations, surveillance, data minimization, and purpose limitation, formed one category. In the same way, the categories were grouped around bias, employment, and social media. Finally, a broader group gathered foundational issues that cut across all other categories and included discussions of AI safety, security, algorithm accountability, artificial moral agents, and the capabilities of the technology.

Thus, we propose five categories: (1) Foundational issues of AI in business; (2) Transparency, privacy, and trust; (3) Bias, preferences, and justice; (4) Employment and automation; (5) Social media, participation, and democracy. We proceeded then to classify each article within one of these.

## Discussion and findings

### Most influential journals (RQ1)

Our group comprises 95 articles published in 54 journals. The *Journal of Business Ethics* (JBE) is the most cited with 1,072 citations; it is also the most productive, with 22 publications. Only five articles were published in JBE between 2000 and 2018 and 17 through 2019 and 2021.

**Figure 1:**
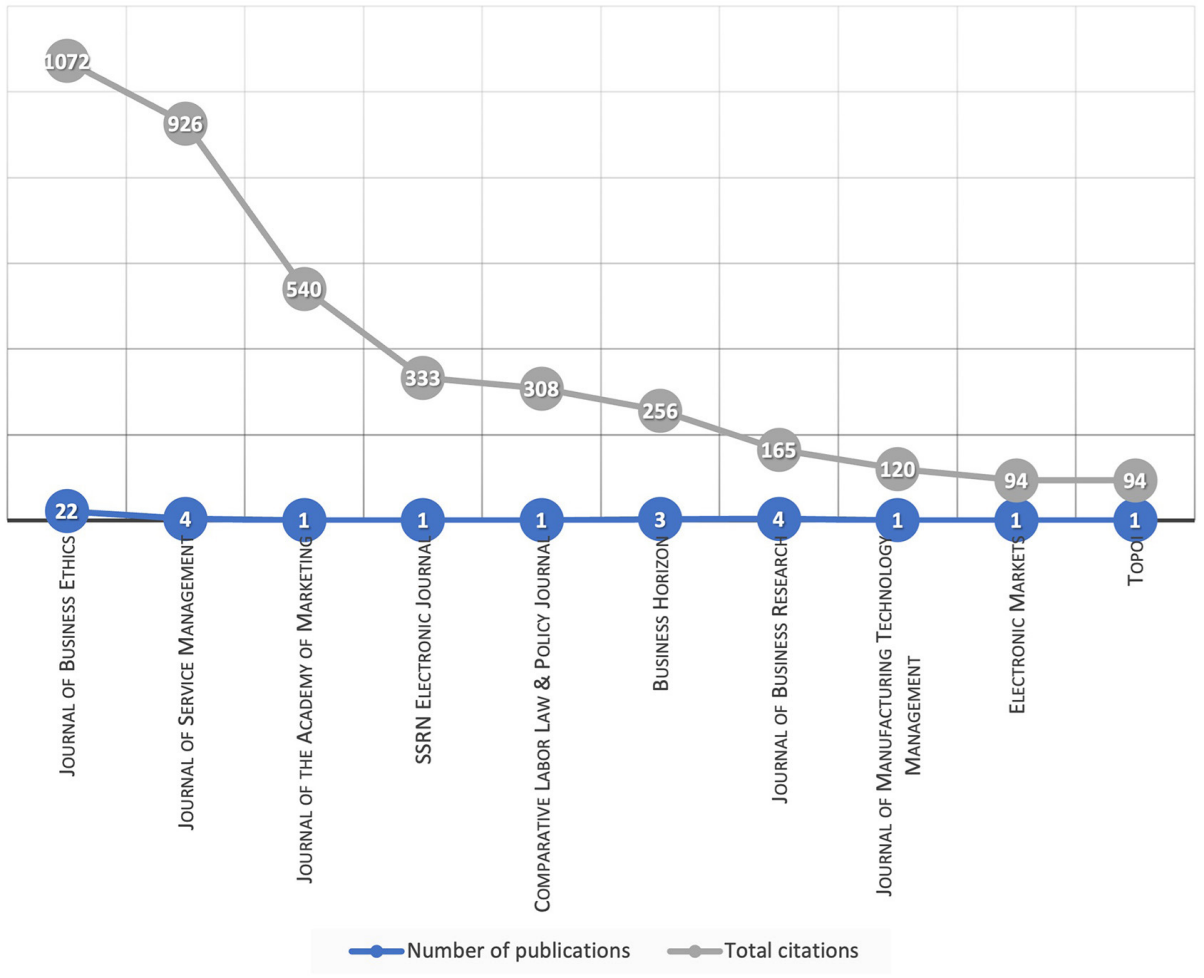
Influence and productivity of academic journals.

JBE is the only journal that addresses all three major schools of ethical thought. The influence of the journal and its broad reach is related to the journal's productivity; between 2000 and mid-2021. JBE published 148 volumes with at least four issues each, while the next most cited journal had only 84 volumes. Figure 1 shows the ten most influential journals by their citations, and the number of publications reflects their productivity.

*Finding 1*: JBE is the most influential and productive journal. It covers a broad range of AI ethics topics and is the only one to address the three major ethical schools.

The *Journal of Service Management* (JSM) followed with 926 citations. However, JSM productivity is far behind with only four articles. The influence of JSM can be explained by one outlier article by Wirtz et al. (2018), which is the most cited in our study, with 734 citations.

Next, the *Journal of the Academy of Marketing Science* (JAMS) is third with 540 citations; its sole publication by Davenport et al. (2020) is the second most-cited article. The fact that a journal with a single publication holds the third position is remarkable. The same case occurs with SSRN with 333 citations in fourth place and Comparative Labor Law and Policy Journal with 308 citations in fifth place, both with only one article. Finally, the rest of the journals obtained less than 300 citations.

*Finding 2*: The most influential journals are specialized in business ethics, management, and marketing.

The *Journal of Business Research* (165 citations) published four articles, all of them in 2021. *Business Horizons* (256 citations) and *Business Ethics Quarterly* (45 citations) had three publications each, one from 2004 and two from 2020. Most journals have only one publication (44 out of 54); however, in some cases, that was enough to position them in the top ten journals, which concentrates 3,908 out of 4,743 total citations.

*Finding 3*: There is an uneven distribution of citations; the top ten concentrates 80%; six journals with only one article are in that list.

### Most influential articles (RQ2)

Our dataset contains 237 authors; the total number of citations is 4,743,^2^ with a mean of 50 citations per article. There is a high concentration in the top five papers, which received 2,199 citations, and only 24 papers have citations above the mean. Table 4 lists the ten most-cited articles with their authors, year of publication, and journal.

**Table 4:** List of 10 most cited articles.

**References**	**Title**	**Citations**	**Source**
Wirtz et al. (2018)	Brave new world: service robots in the frontline	734	Journal of Service Management
Davenport et al. (2020)	How artificial intelligence will change the future of marketing	540	Journal of the Academy of Marketing
Cappelli et al. (2019)	Artificial intelligence in human resources management: challenges and a path forward	333	SSRN Electronic Journal
Cherry (2016)	Beyond Misclassification: The Digital Transformation of Work	308	Comparative Labor Law and Policy Journal
Martin (2019)	Ethical Implications and Accountability of Algorithms	284	Journal of Business Ethics
Kaplan and Haenlein (2020)	Rulers of the world, unite! The challenges and opportunities of artificial intelligence	169	Business Horizon
Johnson (2015)	Technology with no human responsibility?	138	Journal of Business Ethics
Garay-Rondero et al. (2020)	Digital supply chain model in Industry 4.0	120	Journal of Manufacturing Technology Management
Leicht-Deobald et al. (2019)	The challenges of algorithm-based HR decision-making for personal integrity	97	Journal of Business Ethics
Thiebes et al. (2020)	Trustworthy artificial intelligence	94	Electronic Markets
Yampolskiy and Fox (2013)	Safety Engineering for Artificial General Intelligence	94	Topoi

Wirtz et al. (2018) published the most influential article with 734 citations, focusing on the impact of service robots in the industry. The most-influential articles focus almost equally on foundational issues and AI's impact on business functions across different industries.

Marketing occupies the top slot of 22 articles and 1,875 citations, almost double that of human resources in second place (see Table 5). The most relevant topics in marketing are customer behavior and sales (Belanche et al., 2020; Davenport et al., 2020; Reshma and Sam Tharakan, 2021; Vlačić et al., 2021), the attention economy, and social media (Bhargava and Velasquez, 2020; Dossena et al., 2020), digital surveillance (Loi et al., 2020), and service robots and chatbots (Wirtz et al., 2018; Henkel et al., 2020; Odekerken-Schröder et al., 2020; Syvänen and Valentini, 2020; Borau et al., 2021; Söderlund and Oikarinen, 2021).

**Table 5:** Business functions addressed in articles.

**Business function**	**Total articles**	**Total citations**
Marketing	22	1,875
Human resources	12	933
Production	8	248
Finance	7	135

Why is marketing the most discussed topic? One reason may be that advertising was the first beneficiary of AI's capabilities. Google applied it to present personalized ads to its users (Zuboff, 2018). Furthermore, McKinsey and Co. considers marketing and sales the area with the most significant potential to benefit from AI, predicting that AI can create $1.4 trillion to $2.6 trillion worth of business value (Chui et al., 2018).

Human resources (HR) followed with 12 articles totaling 933 citations. The discussions on technological unemployment and automation (Sutton et al., 2018; Kim and Scheller-Wolf, 2019; Holford, 2020; Beerbaum and Otto, 2021), digital transformation, and the devaluation of work (Cherry, 2016; Rodriguez-Lluesma et al., 2020), new competencies and future skills (Moldenhauer and Londt, 2019; Leitner-Hanetseder et al., 2021) and algorithm-based HR decisions (Leicht-Deobald et al., 2019; Terblanche, 2020), are relevant to this topic.

Third was production with eight articles and 248 citations, and finance was fourth with seven articles and 135 citations. Here, the supply chain (Garay-Rondero et al., 2020), technology design and development (Neubert and Montañez, 2020; North-Samardzic, 2020; Ryan and Stahl, 2021), auditing (Munoko et al., 2020), accounting (Losbichler and Lehner, 2021), and taxes (Berger et al., 2020; LaMothe and Bobek, 2020), among other issues, are addressed.

Robotics and RPA have optimized many processes in finance and production with substantial effects on cost reduction, though it may have caused job losses and devaluation of human work. Despite existing dilemmas, the study of ethical issues in both seems to be a research area under development.

*Finding 4*: Marketing dominates among business functions, followed by human resources, production, and finance.

The foundational issues cut through many domains. These articles address AI's current and future capabilities (Kaplan and Haenlein, 2020), machines' autonomy to make decisions (Johnson, 2015), reliability and accountability of algorithms (Martin, 2019), and how to develop safe and trustworthy AI (Yampolskiy and Fox, 2013; Thiebes et al., 2020). Other issues of concern include employment and the devaluation of work, privacy violation, algorithmic bias, and the effects of social media on society.

#### An explosive increase in interest in the ethics of AI in business

**Figure 2:**
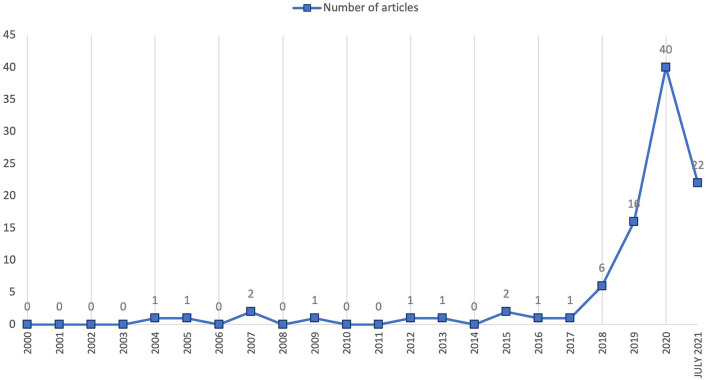
Number of publications between 2000 and mid-2021.

Between 2000 and 2017, there were only 11 publications on the ethics of AI in Business (see Figure 2). An explosive increase in publications followed; 84 articles were published between 2018 and 2021. Twenty years ago, there was less research production, digital publications were less frequent, and open access was less extensive.

**Figure 3:**
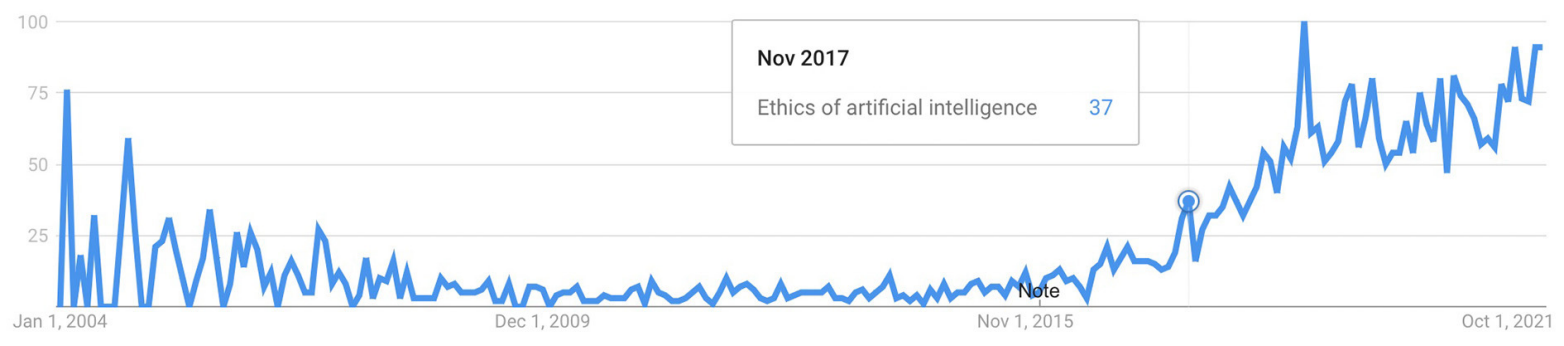
Interest over time in “Ethics of artificial intelligence.” Source: Google trends.

Most citations belong to papers published from 2018 onwards, coinciding with the increase in scientific publications. Thus, it is consistent with the increase in Google searches on the term “ethics of AI” (see Figure 3).

**Figure 4:**
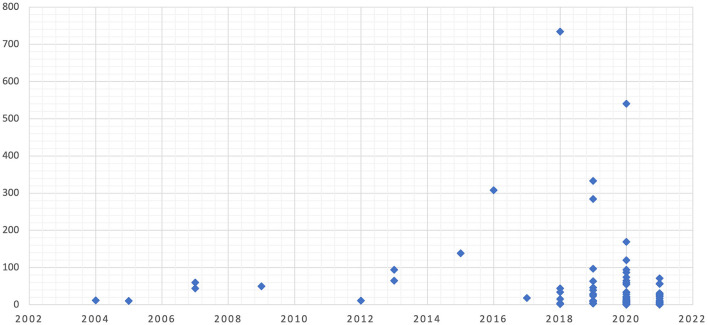
Citations by year of publication.

Although generally, the older an article, the greater the chances of being cited; in this case, the most cited articles were published in the last four years, as shown in Figure 4. There is one exception, “Beyond Misclassification: The Digital Transformation of Work,” with 308 citations by Cherry (2016). This article is the first to address one of the ethical issues in a factual and not merely conceptual way, referring to the impact of this technology on the labor market.

Cherry (2016) analyzes the transformation of work through different labor court cases in the on-demand economy. *Crowdwork* has promoted the proliferation of precarious work, which includes automatic management and workers' deskilling, offering a disturbing image of future work.

One possible reason for this article's influence is that it is the first to present evidence of the harm that AI could cause in labor. Before Cherry (2016), issues addressed were more hypothetical than factual. Concerns revolved around what might happen if the technology gained new capabilities. Subsequent publications deal with real issues and situations affecting people.

#### A change in conversation: From objects to subjects

Early publications focused not on AI but on moral issues related to technology's impact on companies. Those publications addressed tensions between proprietary and open-source software (Schmidt, 2004), the misuse of IT resources within the workplace (Chu et al., 2015), and whether computers can help make better ethical decisions (Mathieson, 2007).

The common denominator is an older conception of AI, resembling “good old-fashioned artificial intelligence” or GOFAI (Grim and Singer, 2020), developed using linear programming. Thus the resulting software was perceived as a tool used for specific purposes with clearly defined rules and limits.

Later publications opened the door to a new conception of AI as a subject. These propose a *moral Turing test* to establish whether corporations (Henriques, 2005) or machines have moral agency (Guarini, 2007) and at which level of intelligence it should be granted (Yampolskiy and Fox, 2013). Johnson (2015) wonders if it is possible that in the future, artificial agents will acquire the capacity for autonomous behavior with no human being responsible for them. As AI became widespread, ethical issues and questions appeared in the scientific literature. Should AI be regarded as natural persons, legal persons, animals, or objects? (Beerbaum and Otto, 2021).

**Figure 5:**
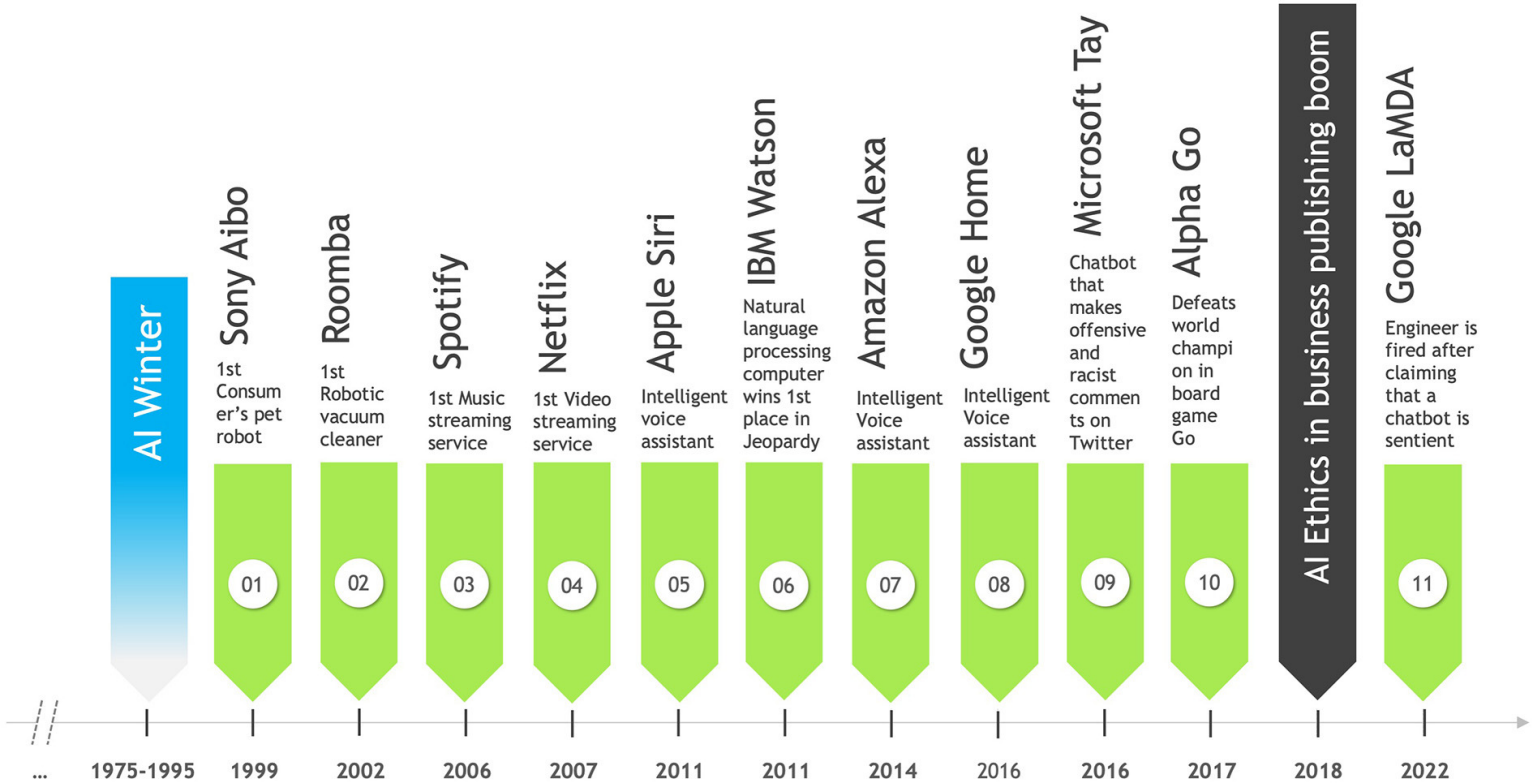
AI in consumer products and services timeline.

After the period of stagnation between 1975 to 1995, known as the “AI winter” (Müller, 2021), the great availability of data, cheaper storage, and new machine learning techniques expanded the applications and capacity of AI. It became more affordable and higher performing entering new spheres. AI ceased to be exclusive to technicians, experts, and scholars and ventured into the market of consumer products and services (see Figure 5).

*Finding 5*: With the incursion of AI into consumer products and services comes an increased interest in the ethics of AI in business in 2018 and a boom in scientific publications.

AI devices were perceived as valuable tools that served people's purposes. Yet there are concerns about how firms handle our data and deal with privacy.

Situations occurred in which machines competed with humans; automation replaced workers and stoked fears that millions of jobs will be lost (Carter, 2018). In 2011, IBM's Watson defeated human champions on Jeopardy (Kaplan and Haenlein, 2020); in 2017, Google's AlphaGo defeated Chinese player Ke Jie^3^ in the game “Go.” A machine that learned the game by playing against itself thousands of times proved to be better than the world champion. Later in 2022, a Google engineer was fired after claiming that LaMDA, a company's chatbot, was sentient and even demanded legal representation for it (Johnson, 2022b).

Kurzweil (2005) claimed that AI would eventually surpass human intelligence, awakening concerns that it will render humans obsolete and useless and, in the worst-case scenario, destroy humanity (Du and Xie, 2021). For Yampolskiy and Fox (2013), “an intelligence that improves itself to levels so much beyond ours that we become not just an ‘inferior race' but destroyed as a side-effect of the entity's activities in pursuit of its goals.”

We believe the increase in publications could be because machines are now perceived as ethical subjects or agents. This technology is capable of mimicking humans (Vlačić et al., 2021), making decisions autonomously, and influencing people and their environment. Concerns arise that AI might pose a threat, and ethics become essential to the conversation.

*Finding 6*: With AI's increased capacity, a change in perception occurs, from AI as an object to AI as a subject or agent; Cherry's (2016) article marks a milestone between scientific publications with hypothetical perspectives and those that address real issues.

### Most influential authors (RQ3)

Based on total citations, we constructed the list of the ten most influential authors (Table 6). In addition, we include the *h-index* to have a second element of comparison to measure the author's influence. This score allows us to measure authors' productivity and impact compared to their total citations; it is calculated using the author's number of publications with at least the same number of citations. Thus, an author with an h-index of 50 has published 50 articles that have been cited at least 50 times. Using the *h-index*, we can eliminate outlier publications that might present a distorted view of an author's impact

**Table 6:** Ten most influential authors by their total citations.

	**Author(s)**	**h-index**	**Citations**	**Institution**	**Country**
1	Davenport et al.	107	123,746	Babson College	USA
2	Grewal et al.	97	75,942	Babson College	USA
3	Haenlein et al.	37	41,737	ESCP Business School, Sorbonne Alliance	France
4	Ferrell et al.	66	40,751	Auburn University	USA
5	Kaplan et al.	36	39,393	ESCP Business School, Sorbonne Alliance	Germany
6	Wirtz et al.	75	37,186	National University of Singapore	Singapore
7	Patterson et al.	54	26,773	University of New South Wales	Australia
8	Jansen et al.	71	26,226	Qatar Computing Research Institute, HBKU	Qatar
9	Chau et al.	60	24,947	University of Nottingham Ningbo	China
10	Capelli et al.	61	22,817	University of Pennsylvania	USA

Furthermore, some authors published most of their work and received most of their citations from previous publications, for instance, in business ethics or management. Therefore, using total citations will measure the author's influence in a broader sense and is not limited to the ethics of AI in business.

The list is dominated by two scholars from Babson College in the US. Davenport has almost twice the number of citations as his colleague Grewal. However, only 10 points separate them in their *h-index*. They co-authored the article “How artificial intelligence will change the future of marketing” (Davenport et al., 2020), which is the second most cited.

Grewal, with 75,942 citations, almost doubles those of the Haenlein. Yet, in this case, the difference between their h-index score is 60. The difference in the number of citations between the top two authors and the rest is noteworthy. From the third position, the differences between the number of citations are not so significant and gradually decrease. However, the h-index scores do not follow the same logic. For example, in the sixth position by its citations, Wirtz has an h-index of 75, the third highest.

*Finding 7*: Davenport from Babson College is the most influential author by its citations. The top ten could change using the *h-index* parameter; Flavian and Roper would substitute Haenlein and Kaplan.

Among the ten most-cited authors, half are marketing professors; two come from management, two from information technologies and computer science, and one from business administration. The predominance of marketing professors corresponds to the findings of RQ2, where we observed that marketing is the most studied domain.

*Finding 8*: Half of the most influential authors are marketing professors.

The most cited works of Davenport, Grewal, O.C. Ferrell, Chau, and Capelli were published before the rise of AI ethics, around 2000, related to management, marketing, and IT. Since then, the first three began the study of AI in business, although only O.C. Ferrell used a specific ethical perspective founded on the deontological and utilitarian schools.

Although they also have relevant works before 2000, Paterson and Jansen published their most influential works around 2010 in marketing and social media. Both continued to research AI in business. Paterson co-authored with Wirtz the most cited article in our dataset in 2018 about AI's foray into the service sector.

The most influential works of Wirtz, Kaplan, and Haenlein were published after 2010. After the arrival of machine learning and deep learning techniques. Their publications' topics are marketing, ethics, and foundational aspects of AI.

The most influential female author, Gaby Odekerken, from Maastricht University, occupies the 12^th^ position with an *h-index* of 34 and 14,242 citations. Men dominate the field; only eleven women are among the 50 most influential authors.

A recent study shows that not the top, but the second and third-tier universities, contributed most to research advances (Fassin, 2022). Our findings bear this out. Only one institution from Shanghai top ten Academic Rankings of World Universities (ARWU) appears in our dataset. The University of Oxford, 7th in ARWU, has Calzada in position 55. Cappelli from the University of Pennsylvania, ranked 15th in ARWU, occupies the tenth position on our list.

Our most influential authors belong to lower-ranked universities, such as Auburn University (ARWU:501-600), National University of Singapore (ARWU:71), and Babson College, ESCP, University of Nottingham Ningbo, Qatar Computing Research Institute, which are not ranked.

*Finding 9*: The most influential authors are affiliated with second and third tier universities and research institutions.

#### The influence of the US and Europe

**Figure 6:**
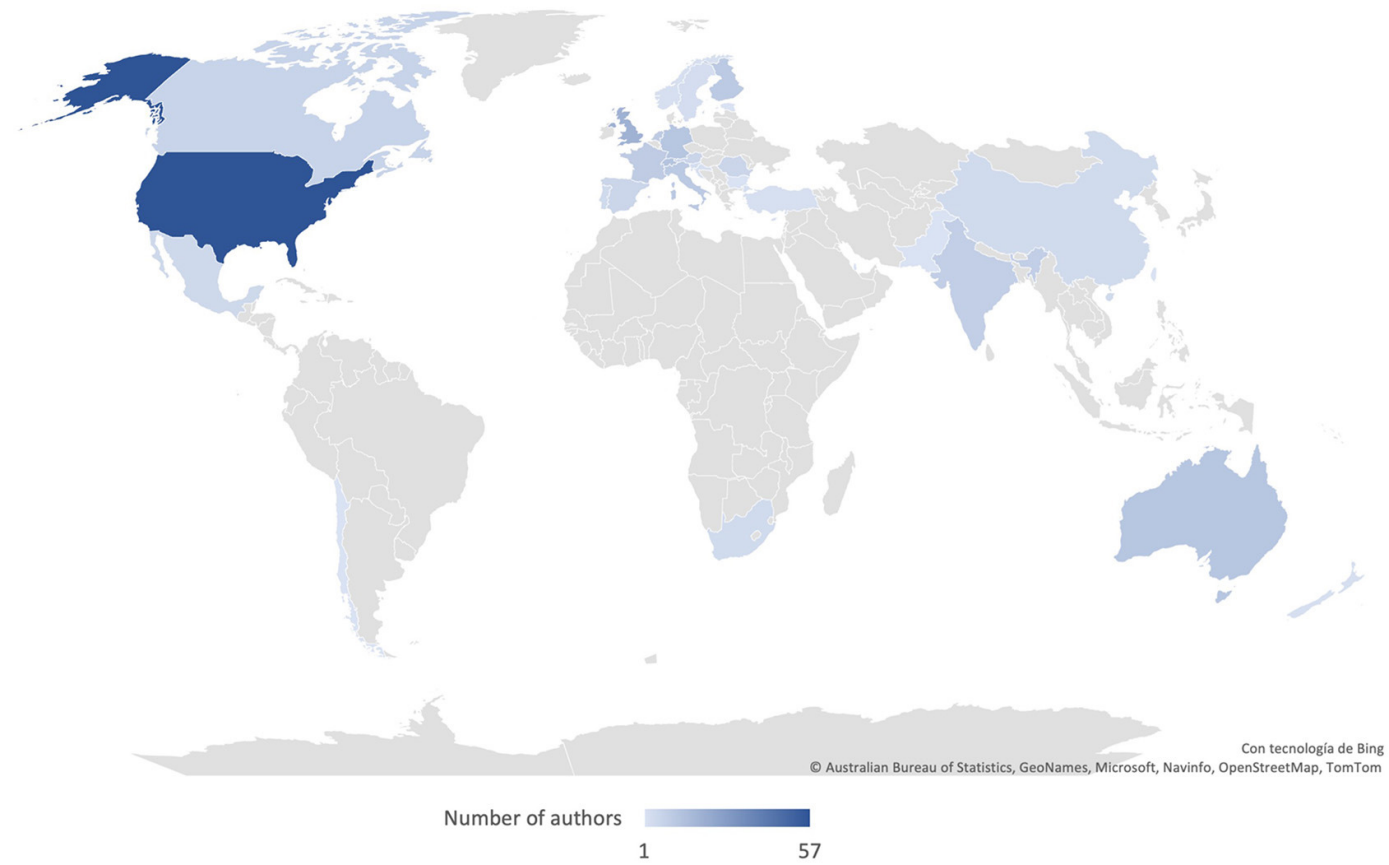
Authors by the country of their institution.

The US leads our group of countries; 57 of 237 authors belong to an American research institution, followed by the UK with 20, Switzerland with 13, and Australia and Germany with 12, respectively. Figure 6 presents a map showing which countries have published the most.

If we consider Europe as a single entity, it would be the most productive, with 126 authors, slightly more than double the US. The high productivity of American and European scholars can relate to the funding available for research and development (R&D). In 2020, it was USD 664 billion for the US (3.4% of its GDP) and 385 for the EU (2.2% of its GDP) (OECD., 2022). However, the US budget is 279 billion higher than the EU, which reflects that the availability of resources is necessary but not decisive; there are other factors.

The world's first legal framework for AI was presented in April 2021 by the European Commission: The Artificial Intelligence Act (AIA). This norm will have a *de facto* effect outside European borders. It is due to the so-called “Brussels effect,” a kind of unilateral regulatory globalization in which EU guidelines become the global market standard (Bradford, 2020).

The construction of legal frameworks closely relates to ethics since it must serve as its foundation. The EU has been more involved in regulating AI than the US and China. Both countries have opted for less regulation, assuming that too much can inhibit innovation and reduce competitiveness (Lee, 2018). This difference could drive or inhibit research in the field.

Asia occupies third place with 19 authors. India contributes with nine; China with six; and Pakistan, Qatar, Singapore, and Taiwan with one each. The small number of Chinese authors is remarkable for a country that in 2020 invested 563 billion in R&D, surpassing the EU. China also surpassed the US in venture capital investment in AI startups in 2017. The Chinese percentage was 48%, almost half of the world's total (Vincent, 2018).

There is a strong push from the Chinese government to encourage the development of AI. Their goal is to make their country the center of global innovation in AI by 2030 (Lee, 2018). For this, they have tried to take advantage of their large population, data wealth, and rapid scalability. The small number of Chinese authors could be because the ethical issues of AI have not raised enough interest due to the lack of political incentives. Also, bear in mind that we included only English publications.

Some events discourage research on the subject. In September 2021, the Chinese government published the country's first AI ethics guidelines (Shen, 2021). This “New Generation of Artificial Intelligence Code of Ethics” was not exempt from criticism. Angshuman Kaushik wrote: “*It is quite mystifying to see a country as infamous as China globally for its AI ethics violations, come up with an Ethics Code for the world to sit up and take notice. Its violations list is endless, ranging from the use of Uighur-tracking facial recognition technology and the use of emotion detection software against them in its Xinjiang province to its flouting of human rights norms and draconian manner of application of the social credit system”* (Montreal AI Ethics Institute., 2022).

The almost null participation of African and Latin American authors is remarkable. Only three countries are represented: South Africa, Mexico, and Chile. We believe that less-developed technology and lack of funding and policies encouraging research and development are among the possible causes.

*Finding 10*: The US and Europe lead in the publication of AI ethics in business articles. However, the productivity of scientific publications on this topic seems to depend not only on funding but the political agenda could also be a factor.

### Major schools of thought for ethics of AI in business (RQ4)

We need ethical theories to better support decision-making and to provide well-founded justifications to act in a determined way. However, there are important incompatibilities among ethical theories. Each has a different approach, and decision processes will not always achieve an ideal; there will be trade-offs (Mathieson, 2007).

The classification of the articles into different ethical theories, or schools of thought, represents a turning point. Only 24 articles use a theoretical approach, and 71 papers do not advocate a specific ethical theory. In the same way, we observe that only six philosophers appear in the list of the 50 most influential authors (by their number of citations); this could be the cause of the few articles that use a specific ethical theory to support their arguments.

We found that publications use three major ethical schools: consequentialist, deontology, and virtue ethics, as shown in Table 7.

**Table 7:** Number of papers and citations according to their ethical theory.

**Ethical theory**	**Number of papers**	**Citations**
Deontological	4	235
Virtue Ethics	7	214
Consequentialist	8	793
Multiple (deontological, consequentialist, and virtue ethics)	5	214
No ethical theory	71	3,287

*Finding 11:* Most AI business ethics authors do not use an ethical theory approach; they lack a philosophical perspective.

Five articles have an eclectic approach. Leicht-Deobald et al. (2019) and Ferrell and Ferrell (2021) observe the differences between deontological and consequentialist perspectives and propose a combination to address AI problems in business.

Letheren et al. (2020) suggest that all three schools should be applied as a lens to decide where ethical dilemmas lie. Mathieson (2007) proposes designing an ethical decision support system using all of them. However, there are often conflicts they do not recognize. Seele et al. (2019) assert that depending on which school of thought is adopted, a given position could lead to contrary assessments.

Personalized pricing can provide an example. Seele et al. (2019) point out that this technology tends to be perceived as unfair, asymmetric, or even inhumane. For instance, Uber taxis charging exorbitant fares during terrorist attacks. It may be appropriate from a deontological perspective since it adheres to its established rules, which seek to attract drivers by increasing prices in places where demand rises. However, from a consequentialist standpoint, it would be questionable, and utterly reprehensible from the view of virtue ethics. Since increasing profit, taking advantage of a dangerous situation does not serve the common good or human flourishing.

*Finding 12*: The preferred ethical theory is consequentialist, followed by virtue ethics, deontology, and eclectic approaches.

Consequentialist approaches dominate our list with eight papers. It is also the most cited, with 793, almost four times as deontological and virtue ethics. This theory states that moral rectitude depends only on the consequences of an act. Consequentialist theories embody the basic intuition that what is best or right is whatever makes the world best in the future (Sinnott-Armstrong, 2021). In this group, we include the *utilitarian* and *behavioral* approaches.

One possible reason for this theory's dominance is that most organizations focus on calculating utility or profits. In business and neoclassical economics, the result is usually privileged over the means. Beerbaum and Otto (2021) uncovers this issue. Using the Uber-Waymo trial as an example, he exposed the culture of agile software development, which prioritizes software release over testing and verification, and encourages shortcuts to diminish costs. Most companies prioritize maximizing quick profits, which is an old issue for business ethics.

The virtue ethics approach is just one article behind consequentialism with seven articles; however, it is third by number of citations. Etymologically, “virtue” comes from the Latin word “virtus,” which stands for “what is best” or “excellence” in human beings. “Virtue,” then, means “what is best in human beings” or “human excellence” (Sison, 2015). Virtue as a framework for ethics differs from rights, duties, and calculations of consequences, and has its focus on good character (Neubert and Montañez, 2020).

Authors who use the virtue ethics approach highlight AI's importance in producing improvements at a societal level and not only to increase profits. Let us examine the effects of addictive algorithms in social media and marketing. Virtue ethics might propose to use practical wisdom such that each person in the design process decides on the extent of user engagement (Thorpe and Roper, 2019). However, this could be problematic as leaving sensitive decisions to people's discretion could lead to inconsistencies or abuse, endangering human flourishing.

Only four articles use an exclusively deontological perspective; however, it is the second most cited. Deontology is a normative duty-based theory that guides and assesses our choices of what we ought to do, in contrast to those that assess what kind of person we are and should be (Alexander and Moore, 2021), such as virtue ethics. Deontologists focus on the action itself and oppose consequentialists who measure the morality of an action based on its consequences. In other words, ethical behavior is based on a predetermined set of norms or rules that must always be followed.

Still, most high-level interventions in the AI ethics discussion are principle-based, such as the guidelines produced by the European High-Level Expert Group on AI (Stahl et al., 2021), IBM's Principles for Trust and Transparency (IBM., 2018), or the Asilomar AI Principles (Future of Life Institute., 2017).

Let us analyze the evolution of ethical theories in the literature. Of all 24 articles in this group, 21 were published between 2019 and 2021, and only three before (see Table 8).

**Table 8:** Articles with an ethical theory perspective.

**References**	**Title**	**Ethical theory**
Letheren et al. (2020)	Black, white or gray magic? Our future with artificial intelligence	Deontological, consequentialist, and virtue ethics
Mathieson (2007)	Toward a design science of ethical decision support	Deontological, consequentialist, and virtue ethics
Seele et al. (2019)	Mapping the Ethicality of Algorithmic Pricing: A Review of Dynamic and Personalized Pricing	Deontological, consequentialist, and virtue ethics
Kaplan and Haenlein (2020)	Rulers of the world, unite! The challenges and opportunities of artificial intelligence	Deontological
Moldenhauer and Londt (2019)	Leadership, Artificial Intelligence, and the need to redefine future skills development	Deontological
Ryan and Stahl (2021)	Artificial intelligence ethics guidelines for developers and users: clarifying their content and normative implications	Deontological
Scharding (2020)	Recognize Everyone's Interests: An Algorithm for Ethical Decision-Making about Trade-Off Problems	Deontological
Ferrell and Ferrell (2021)	Applying the Hunt Vitell ethics model to artificial intelligence ethics	Deontological and consequentialist
Leicht-Deobald et al. (2019)	The challenges of algorithm-based HR decision-making for personal integrity	Deontological and consequentialist
Borau et al. (2021)	The most human bot: Female gendering increases humanness perceptions of bots and acceptance of AI	Consequentialist: utilitarian
Clarke (2019)	Principles and business processes for responsible AI	Consequentialist: utilitarian
Hermann (2021)	Leveraging Artificial Intelligence in Marketing for Social Good - An Ethical Perspective	Consequentialist: utilitarian
Kriebitz and Lütge (2020)	Artificial intelligence and Human rights: a business ethical assessment	Consequentialist: utilitarian
Odekerken-Schröder et al. (2020)	Mitigating loneliness with companion robots in the COVID-19 pandemic and beyond: an integrative framework and research agenda	Consequentialist: utilitarian
Beerbaum and Otto (2021)	Artificial Intelligence Ethics Taxonomy - Robotic Process Automation (RPA) as Business Case	Consequentialist: behavioral
Chu et al. (2015)	Explaining the Misuse of Information Systems Resources in the Workplace	Consequentialist: behavioral
Davenport et al. (2020)	How artificial intelligence will change the future of marketing	Consequentialist: behavioral
Henkel et al. (2020)	Robotic transformative service research: deploying social robot for consumer well-being during COVID 19 and beyond	Virtue ethics
Kim and Scheller-Wolf (2019)	Technological unemployment, meaning in life, purpose of business, and the future of stakeholders	Virtue ethics
Neubert and Montañez (2020)	Virtue as a framework for the design and use of artificial intelligence	Virtue ethics
Thorpe and Roper (2019)	The ethics of gamification in a marketing context	Virtue ethics
Schmidt (2004)	Intellectual Property Battles in a Technological Global Economy: A Just War Analysis	Virtue ethics
Stahl et al. (2021)	Artificial intelligence for human flourishing – Beyond principles for machine learning	Virtue ethics
Trinh and Castillo (2020)	Practical wisdom as an adaptive algorithm for leadership: Integrating Eastern and Western perspectives to navigate complexity and uncertainty	Virtue ethics

The first three articles were published between 2004 and 2015. Schmidt (2004) alludes to the natural law theory approach associated with virtue ethics. He examines the conflicts that arise over intellectual property and software licenses. Mathieson (2007) studies the use of a support system for ethical decision-making. And Chu et al. (2015) use behavioral theory, related to the consequentialist approach, to explain the reasons for information systems resources misuse in the workplace.

The topics covered in these first three articles bear little relation to the current conception of AI perceived as a subject. At this stage, most machines are objects with no autonomy and limited capacity. The ethical responsibility for ethical issues rests solely with the users of the technology, just as it would with a knife which can be used both as a tool or a weapon.

As of 2019, AI-driven machines capable of autonomous learning with predicting and decision-making capacity have become widespread. As most papers were published in the last three years, it is hard to establish any trend.

An assessment of the benefits and harms caused by AI marks later publications. Tradeoffs will have to be made, evidencing the need for ethical judgment. Moral questions appeared; are the algorithms unbiased, impartial, and efficient? (Leicht-Deobald et al., 2019); who will be responsible for the ethical consequences of decisions made by algorithms? (Martin, 2019).

Amazon discovered that its AI hiring algorithm discriminated against women and had to drop its use. Even when the sex of applicants was not being used as a criterion, attributes associated with women candidates caused them to be ruled out (Cappelli et al., 2019). The reason was that the training datasets were based on previous applicants, predominantly men (Davenport et al., 2020). Martin et al. (2019) propose that if a design team creates an impenetrable AI decision, then the firm should be responsible for those decisions.

In later publications, not just the user of the technology could be held accountable but also organizations, firms, and developers (Belanche et al., 2020) who sometimes try to hide behind the opacity of algorithms (Martin, 2019; Carroll and Olegario, 2020).

*Finding 13*: Initially, accountability was attributed exclusively to the user; later, it was extended to developers and firms.

### Main ethical issues of AI in business (RQ5)

Multiple ethical issues appeared as AI acquired greater power and complexity. These issues cover a broad spectrum, from privacy violations to world domination by sentient machines. However, we will not focus on the dangers of AI acquiring consciousness and will of its own since we consider this more fictional than factual.

**Figure 7:**
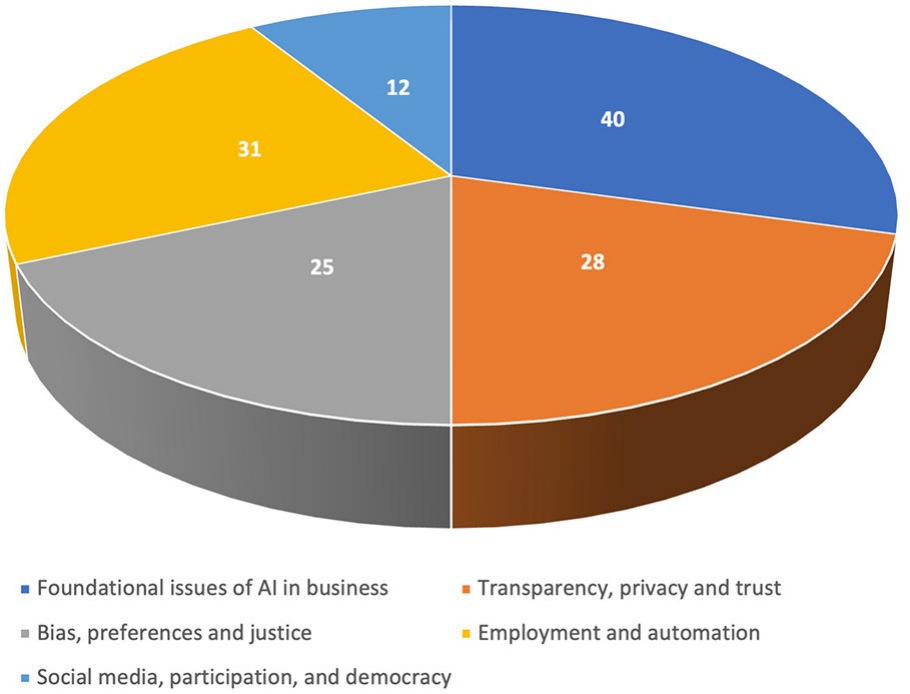
Main ethical issues of AI in business.

This section describes our findings regarding the main issues in the business AI ethics literature. It is organized according to the five categories we built from analyzing the problems, concerns, and values we identified around the main debates. Figure 7 shows this classification exercise.

*Finding 14*: Five categories can group the main ethical issues of AI in business: 1) foundational issues of AI in business; 2) transparency, privacy, and trust; 3) bias, preferences, and justice; 4) employment and automation; and 5) social media, participation, and democracy.

#### Foundational issues of AI

These articles focus on the comprehensive characteristics of the technology, its capacities, possibilities, and technical aspects. This category intersects with the other four identified. We find references to the three levels of intelligence that AI can possess. The first two are Artificial Narrow Intelligence (ANI) and Artificial General Intelligence (AGI); both can equal or outperform human performance. Though ANI is focused on a specific domain and AGI can extend into new domains (Davenport et al., 2020).

There is currently no functional AGI. However, once an AI with that ability is created (if at all), it could improve its ability using machine learning. At some point, it could surpass human levels and increase its intelligence exponentially without stopping. This intelligence explosion is known as *singularity* and would result in Artificial Super Intelligence (ASI). ASI is a hypothetical group of self-aware systems capable of scientific creativity, social skills, and general wisdom (Kaplan and Haenlein, 2020).

A significant challenge to the claim that only human beings can be responsible comes from those for whom agents can learn as they operate (Johnson, 2015). However, all existing AIs are below human levels of intelligence, and we generally do not ascribe moral agency to infrahuman agents such as non-human animals or even children (Yampolskiy and Fox, 2013). Therefore, humans should be held accountable for AI's negative impacts or harms.

Some authors propose principles, guidelines, and frameworks to avoid risks and mitigate possible damages (Cole and Banerjee, 2013; Yampolskiy and Fox, 2013; Clarke, 2019; Kriebitz and Lütge, 2020; Neubert and Montañez, 2020; Ferrell and Ferrell, 2021). Others explore specific problems and propose solutions, like Fischer et al. (2021), who suggests using this technology to combat climate change. Thus, the discussion about the responsible development and deployment of AI appears.

Another foundational debate is that of ethical decision-making with the help of AI. Unethical behavior in business can harm companies and make their employees personally liable (Mathieson, 2007), with economic, legal, and social consequences. AI-enabled decision support systems have sought to deliver timely and reliable information to decision-makers. However, these systems' biases have caused discrimination and unfairness. Additionally, the perception that these systems are more efficient and free of bias has led to excessive confidence and, in some cases, to delegate full responsibility to them.

#### Transparency, privacy, and trust

AI needs large amounts of data to perform tasks and expand capabilities. However, collecting this data could conflict with the right to privacy (Kriebitz and Lütge, 2020), as it is often obtained without user consent. Furthermore, AI-enabled systems can perform sophisticated tasks like biometric and facial recognition or natural language processing, enabling unprecedented surveillance techniques.

Privacy and transparency are recurrent issues in business functions, such as marketing and sales (Thorpe and Roper, 2019; Hermann, 2021). Companies like Google, Amazon, and Facebook use people's personal information for targeted advertising (Kaplan and Haenlein, 2020). The tension between privacy and transparency presents a dilemma for users of digital platforms. When browsing the Internet or using a smartphone, we generate information about our habits and preferences, which are then stored and later (or immediately) used to predict or influence our behavior (Guha et al., 2021).

However, they are not the only domains where privacy is relevant. Algorithmic HR decision-making requires employee monitoring, often without their knowledge (Leicht-Deobald et al., 2019). Furthermore, companies that use algorithmic pricing, such as insurers, ridesharing, or airlines, require access to personal data (Seele et al., 2019), which could lead to discrimination. Another example is the application of AI in the interrogation tools of judicial systems, such as *facial sentiment analysis*, where the legal principle of *nemo tenetur se ipsum accusare*, no one can be forced to accuse himself, would be violated (Kriebitz and Lütge, 2020).

Additionally, AI-powered devices such as drones, doorbells, or surveillance cameras in shops store information in the cloud. Customers become concerned if companies have access to data they could use or sell. Neighbors might protest if cameras record their front yard activities without permission. Also, the data could be subpoenaed by law enforcement agencies or obtained illegally by hackers (Davenport et al., 2020).

Data breaches and theft of sensitive information are troubling, but the possibility of being used by an autocratic government against its people represents a more significant concern. The Chinese government uses facial recognition technology to monitor its citizens within its social credit system (Calzada and Almirall, 2020), which has been used to oppress Uyghur Muslims in Xinjiang province (Kriebitz and Lütge, 2020).

Furthermore, AI-driven devices can classify people based on age, gender, race, or sexual orientation (North-Samardzic, 2020). Researchers from Cambridge University and Microsoft were able to predict sexual orientation with only a few Facebook likes, with an 88% accuracy in men and 75% in women (Rosen, 2013). The ease of obtaining these predictions could raise concerns when considering that there are still eleven countries that criminalize LGBT people and can impose the death penalty^4^

#### Bias, preferences, and justice

The criteria used by machines for decision-making are not always clear and constitute a *black box* (Kaplan and Haenlein, 2020). On many occasions, this information is protected by business secrecy; at other times, it is impossible or too expensive to isolate which exact factors these algorithms consider (Davenport et al., 2020).

Google's AI language translation algorithm produced gender-biased results in the Turkish language. In translating a gender-neutral pronoun, the algorithm decided that men would be described as entrepreneurial while women were described as lazy (Neubert and Montañez, 2020).

Another emblematic case is Tay, Microsoft's AI-enabled chatbot (see Figure 5), which learned by screening Twitter feeds and took less than 24 hours to publish politically incorrect messages full of misogyny, racism, pro-Nazi, and anti-Semitic (Kriebitz and Lütge, 2020). Indeed, the machine itself was not racist but learned racism from our previous behavior. This gives us a disturbing picture of how other AI-enabled systems might operate now or in the future.

AI-system biases have the veneer of objectivity, yet the algorithm created by machine learning can be just as biased and unjust as one written by humans (Martin, 2019). Worse, given their rapid proliferation in businesses and organizations, AI systems can reproduce and amplify these biases exponentially and cause serious harm.

In 2016, a ProPublica investigation found that software used in some US courts to assess the potential risk of recidivism discriminated against racial minorities. This program called Correctional Offender Management Profiling for Alternative Sanctions (COMPAS) returned scores in which blacks were almost twice as likely to be labeled as higher risk but not actually re-offend (Angwin et al., 2016). Decisions made under the influence of this algorithm can have severe repercussions. Not only is it a matter of getting out on parole, but a criminal record can make it challenging to get a job in the future.

The damage caused by algorithm discrimination may not be deliberate. However, this does not mean that the company and the developers of the biased technology should not be held accountable. Acknowledging bias has led to calls for algorithms to be “explainable” or “interpretable” (Martin et al., 2019).

#### Employment and automation

The deployment of AI in all business areas came with a paradigm shift in the labor market. It is the second most frequent topic in our study and emerges as one of the biggest concerns, with 31 articles addressing it. Three main topics appear, the proliferation of precarious jobs in the *On-Demand Economy* (the *gig economy*), the replacement of humans in work, and the loss of jobs due to automation.

Let us review the case of platforms such as *Uber, Lyft, Crowdflower, TaskRabbit*, and other *On-Demand Economy* companies that built their business model by putting people in contact for micro-tasks. This model is also known as “*crowdwork*,” and contrary to what is happening with robots and RPA, it has fueled the proliferation of new jobs. However, this trend is associated with transient and non-linear careers and has devalued work, promoting wages below the legal minimum and becoming an excuse to avoid paying social security benefits (Cherry, 2016; Rodriguez-Lluesma et al., 2020).

Furthermore, RPA has become a significant trend (Beerbaum and Otto, 2021) due to its ability to operate uninterruptedly, with high scalability and low operating costs. It is the software equivalent in offices to mechanical robots in factories and has rapidly replaced humans in different fields. This phenomenon accelerated during the COVID-19 pandemic due to confinement measures. A consequence is that many jobs have been lost, albeit in subtle ways. Although most robots are not physically replacing workers by taking over their desks, many of these job losses are positions that were handled by individuals or those of companies that went bankrupt. For instance, the explosive growth of streaming video platforms like Netflix caused companies like Blockbuster to close; many small bookstores and retailers closed, and their jobs were taken over by Amazon's 200,000 robots (Roose, 2021; Koetsier, 2022).

Notwithstanding, automation sometimes does not constitute an innovation or an improvement for efficiency; it simply mimics what a human does, for example, in self-checkout kiosks. This phenomenon, referred to as “so-so automation” (Acemoglu et al., 2022), does not lead to value and wealth creation but only to job losses and the devaluation of work.

Nevertheless, some authors believe that fears of AI leading to mass unemployment are unlikely. They argue that new industries will emerge, creating more jobs than lost (Autor, 2015; Kaplan and Haenlein, 2020; Malone et al., 2020; Rodriguez-Lluesma et al., 2020; Beerbaum and Otto, 2021). Yet, nobody knows if newly created jobs will be enough or when it will happen.

We observe that the impact of AI on the labor market has ambivalent implications. These changes represent a challenge that, if not addressed correctly, could accentuate income inequality between individuals and social classes. Part of this discussion revolves around ensuring that the new wealth is distributed fairly and equitably, including those who will be left jobless. While some authors propose that machines and humans should collaborate instead of competing, we agree that AI would be more effective if focused on increasing the capabilities of humans instead of replacing them (Sutton et al., 2018; Davenport et al., 2020; Guha et al., 2021; Brynjolfsson, 2022).

#### Social media, participation, and democracy

For some, AI-enabled social media is a support tool for business functions, for example, in sales (Reshma and Sam Tharakan, 2021), marketing (Dossena et al., 2020), customer service (Murtarelli et al., 2021), management (Delanoy, 2020), and public relations (Rantanen et al., 2020). However, we will focus on the societal repercussions of social media.

Unlike most businesses where the product is the source of income, on social media platforms, the users' attention is sold as a product to advertising companies (Bhargava and Velasquez, 2020). In a model called the attention economy, the services of, for example, Google, TikTok, or Facebook are designed to keep users engaged as long as possible. The longer users stay, the more the companies earn by offering relevant, user-targeted ads based on their habits, mood, or purchase intentions.

According to Bhargava and Velasquez (2020), these companies use “adaptive algorithms” to personalize the content and ads appearing in an endless user feed, causing an addiction already recognized as a public health problem in some countries. Kaplan and Haenlein (2020) observe that excessive use of social platforms may be associated with increased anxiety and depression. They observe other problems of social media, such as the dissemination of fake news, cyberbullying, and harassment.

Finally, some authors remark that social media platforms are used by hate activists to propagate messages that produce strong emotions against victims. Rauf (2021) considers getting caught in the debate easily, even for critics of such hate. It leads to a vicious cycle that provides data for social media companies, garners more publicity for the topic, and attracts others to it. In his article, Rauf depicts social media as an enabler of terror before, during, and after the 2019 Christchurch terrorist attacks in New Zealand.

*Finding 15*: Initial papers addressed foundational issues only. After 2016, issues around privacy, bias, employment, and social media's effect on society appeared.

## Conclusion

This work presents an overview of the most influential journals, articles, and authors in literature. It allows us to understand the current state of publications on AI ethics in the field of business broadly and comprehensively; our first and second motivations are thus satisfied. However, the small number of articles that frame arguments from some of the main ethical schools of thought has made it challenging to connect the main issues with the main ethical theories.

In this work, a map describes how the conceptual space is distributed in terms of a journal, article, or author influence and the prominence of an ethical issue or school. A trend describes how the distribution of that conceptual space varies over time. Our findings allowed us to draw maps and trends formulated through the following propositions.

**Proposition 1 (map)**: JBE is the most influential (by number of citations), productive (by number of articles), and comprehensive (by breadth of topics and schools) journal; although other journals published the top three most cited articles.

**Proposition 2 (trend)**: JBE is the most consistent journal publishing articles from 2000 to 2021.

**Proposition 3 (map)**: The most influential articles (by number of citations) are distributed almost equally among business functions and foundational issues. Among the business functions, the top slot belongs to marketing, followed by human resources, and production and finance afterward. The foundational issues discuss AI's current and future capabilities, accountability, and trustworthiness.

**Proposition 4 (trend)**: Hardly any articles were published until 2018, when there was an explosion. Possible causes are a) the beginning of the widespread use of consumer AI (enabled by greater availability of data, cheaper data storage, and machine learning techniques) and b) the shift in perception from AI as object or tool to AI as subject or agent that can compete or even supplant humans. What before was a mere hypothesis now becomes an imminent possibility.

**Proposition 5 (map)**: Davenport and Grewal from Babson College in the US are the most influential authors on the ethics of AI in business. The ten most influential authors are male, and half are marketing professors. We observe a dominance of authors affiliated with US and EU institutions, and China's absence is notable given its government's manifest interest in taking a leading role in AI development.

**Proposition 6 (trend)**: Most influential authors had a solid research record even before the AI ethics in business boom in 2018. Their research on AI ethics is an extension of their previous works.

**Proposition 7 (map)**: Most authors (71) do not use an ethical theory to support their positions on the ethics of AI in business. However, among those who do use a school of thought, consequentialists (8) dominate, closely followed by virtue ethics (7) and deontology (4), and there are five that use a combination of them. The small number of articles with an ethical theory approach makes the connection between AI ethics and other, more comprehensive ethical domains more difficult.

**Proposition 8 (trend)**: Almost all articles using an ethical theory were published after 2019; only three are previous. The first articles placed the responsibility for the outputs of the technology exclusively on the user. After the adoption of consumer AI and the shift to understanding AI as a subject or agent, articles deal with AI, and the firms and developers are added as accountable instances.

**Proposition 9 (map)**: Foundational issues are the dominant category; they cut across different domains and are usually combined with other topics. Next is employment and automation, perhaps where the harms and benefits caused by AI are most immediate. However, privacy violations, algorithmic bias, and social media's effects follow closely, where harms are probably perceived as less severe.

**Proposition 10 (trend)**: Work of Cherry (2016) marks a turning point between the hypothetical and the factual approaches in articles. And although the distribution of foundational issues papers covers the entire range of years, all works published before 2016 were within its domain. Subsequent works deal with issues such as privacy, bias, employment, and social media's effects on social participation.

AI ethics in business is a growing research field. We propose a future research agenda to deepen our findings and verify some of our hypotheses.

- First, we think further research is needed to verify if the results obtained in this current study apply to domains of AI ethics other than business, for example, political science, computer science, or medicine.
- Furthermore, we believe further studies are needed to measure the impact of the political agenda on the productivity of scientific articles in Europe, the US, and China. In the same way, researchers could verify the hypothetical reasons we offer to explain the 2018 AI ethics in business publications boom.
- This work found that few articles explored AI ethics from a philosophical perspective; this represents an opportunity, particularly in production and finance, which are currently under-researched areas. Our findings suggest that authors with more profound philosophical training tend to use ethical theories as a foundation in their articles; further research is needed to verify this hypothesis.
- The small number of articles using an ethical school of thought in their arguments made it hard to establish connections between schools and specific issues. Future research is needed to close this gap. Additionally, a contrast with the findings of this work can be established from the study of the most influential issues and ethical schools in Chinese publications.

The study of the ethics of AI could contribute to developing technology at the service of humans and aspire to create value, provide well-being for society, and promote the supreme good and final end of human life: happiness (Sison, 2015).

## Data availability statement

The original contributions presented in the study are included in the article/supplementary material, further inquiries can be directed to the corresponding author.

## Author contributions

MD analyzed the dataset and classified articles according to the ethical schools of thought and main issues, prepared the graphs and tables of the study, and carried out the analysis of the results and the identification of findings and final propositions. UI designed and executed the search strategy, the dataset analysis, and classified articles according to the ethical schools of thought and the main issues and participated in the drafting of the document. Both the authors contributed to the article and approved the submitted version.

## Footnotes

^1^Largest Companies by Market Cap (2022, May 20). https://companiesmarketcap.com

^2^Citations updated for each article on May 11^th^ of 2022.

^3^AlphaGo (2022, April 10). https://www.deepmind.com/research/highlighted-research/alphago

^4^ https://www.humandignitytrust.org
